# Quantitative phosphoproteomic analysis of prion-infected neuronal cells

**DOI:** 10.1186/1478-811X-8-28

**Published:** 2010-09-28

**Authors:** Wibke Wagner, Paul Ajuh, Johannes Löwer, Silja Wessler

**Affiliations:** 1 Paul Ehrlich Institute, Paul Ehrlich-Straße 51-59, D-63225 Langen, Germany; 2 Dundee Cell Products Ltd, James Lindsay Place, Dundee Technopole Dundee, DD1 5JJ, UK; 3 Division of Microbiology, Paris-Lodron University, Salzburg, Austria

## Abstract

Prion diseases or transmissible spongiform encephalopathies (TSEs) are fatal diseases associated with the conversion of the cellular prion protein (PrP^C^) to the abnormal prion protein (PrP^Sc^). Since the molecular mechanisms in pathogenesis are widely unclear, we analyzed the global phospho-proteome and detected a differential pattern of tyrosine- and threonine phosphorylated proteins in PrP^Sc^-replicating and pentosan polysulfate (PPS)-rescued N2a cells in two-dimensional gel electrophoresis. To quantify phosphorylated proteins, we performed a SILAC (stable isotope labeling by amino acids in cell culture) analysis and identified 105 proteins, which showed a regulated phosphorylation upon PrP^Sc ^infection. Among those proteins, we validated the dephosphorylation of stathmin and Cdc2 and the induced phosphorylation of cofilin in PrP^Sc^-infected N2a cells in Western blot analyses. Our analysis showed for the first time a differentially regulated phospho-proteome in PrP^Sc ^infection, which could contribute to the establishment of novel protein markers and to the development of novel therapeutic intervention strategies in targeting prion-associated disease.

## Findings

Transmissible spongiform encephalopathies (TSEs) are fatal neurodegenerative diseases occurring in many different host species including humans, which develop *e.g*. Creutzfeld Jacob disease (sCJD) [1]. The development of TSEs is associated with the self-propagating conversion of the normal host cellular prion protein (PrP^C^) into the abnormal protease-resistant isoform (PrP^Sc ^or PrP^res^) in an autocatalytic manner [2]. PrP^Sc ^plays a key role as an infectious agent in certain degenerative diseases of the central nervous system [3].

The cellular functions of PrP^C ^and PrP^Sc ^still remain enigmatic. The cellular prion protein can be variably glycosylated at two N-glycosylation sites and is C-terminally attached to the cell surface by a glycosyl phosphatidylinositol (GPI) anchor. GPI-anchored proteins are found in lipid rafts, highly cholesterol- and glycolipid-enriched membrane domains associated with a large number of signaling molecules such as G-protein-coupled receptors and protein kinases suggesting that signaling transduction pathways might play a role in TSEs [4]. Hence, previous publications described a functional role of PrP^C ^as a signaling molecule with major findings indicating that PrP^C ^interacts with and activates Src family kinases [5-7]. Increased levels of active Src kinases in scrapie-infected cells then led to the activation of downstream signal transduction pathways [8]. Recently, activation of the JAK-STAT signaling pathway in astrocytes of scrapie-infected brains was observed underlining that signal transduction pathways may play pivotal roles in prion pathogenesis [9]. Interestingly, it was demonstrated that inhibition of the non-receptor tyrosine kinase c-Abl strongly activates the lysosomal degradation of PrP^Sc ^[10]. These data indicate that specific interference with cellular signaling pathways could represent a novel strategy in treatment of TSEs.

**Figure 1:**
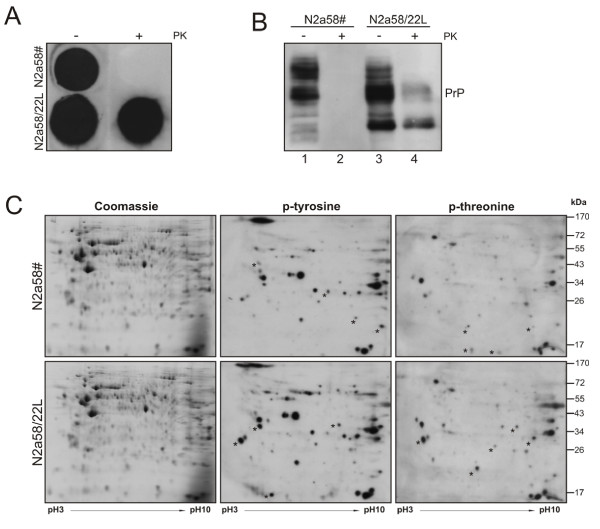
**Differentially phosphorylated proteins in PrP^Sc^-positive and -negative N2a cells**. **(A) **PrP^res^-positive N2a58/22L cells were treated with pentosan polysulfate (PPS) to obtain PrP^res^-negative N2a58# cells. Successful PPS treatment was validated in a colony assay. Cells were grown to confluence on cover slips and directly lysed on nitrocellulose. Where indicated 20 μg/ml proteinase K (PK) was added followed by the detection of PrP expression using the 6H4 monoclonal antibody. In non-treated cells (-), PrP was detected in both, cured and infected N2a cells. Upon digestion with PK (+), PrP^res ^was only observed in N2a58/22L cells. **(B) **Equal amounts of protein lysates were incubated with 20 μg/ml PK or left untreated. PrP was detected with the 8H4 monoclonal antibody showing the typical migration pattern of PrP and PrP^res ^in infected and PPS-treated N2a58# cells. In parallel, lysates were incubated with PK to visualize PK-resistant PrP^res ^in N2a58/22L. **(C) **150 μg of N2a58# or prion-infected N2a58/22L cell lysates were separated by two-dimensional gel electrophoresis followed either by Coomassie staining or immunoblotting for detection of tyrosine- and threonine-phosphorylated proteins. Black asterisks indicate changed intensities of protein phosphorylation.

We have performed a quantitative analysis of the phospho-proteome to obtain a global insight into deregulated signal transduction pathways in scrapie-infected neuronal cells. We analyzed tyrosine- and threonine-phosphorylated proteins in the murine neuroblastoma cell line N2a58/22L, which were infected with the PrP^Sc ^strain 22L [11]. We have treated N2a58/22L cells with pentosan polysulfate (PPS), a known inhibitor of 22L PrP^Sc ^replication in N2a cells [12], resulting in the PrP^Sc^-rescued cell line N2a58# which served as an uninfected control. Successful rescue from PrP^Sc ^was demonstrated in the colony assay as reflected by the absence of proteinase K (PK)-resistant PrP^Sc ^in N2a58# cells after PPS treatment (Figure 1A). PrP^Sc ^replication and the effect of PPS-treatment were further studied in an immunoblot. After PK digestion, PrP^Sc ^replication was only observed in N2a58/22L cells (Figure 1B, lanes 2 and 4). Compared to 22L-infected N2a58/22L cells, PPS-treated N2a58# cells showed a different glycosylation profile as expected for PrP^C ^[13-15]. The glycosylation pattern of PrP^C ^in N2a58# cells displayed high amounts of di- and mono-glycosylated PrP^C^, whereas in N2a58/22L cells predominantly mono- and non-glycosylated PrP^Sc ^was detected (Figure 1B, lanes 1 and 3). Altogether, PPS treatment of N2a58/22L cells successfully abolished PrP^Sc ^formation in N2a58# cells, which served as a non-infected control cell line in our study.

To analyze differentially phosphorylated proteins in N2a58/22L cells in comparison to N2a58# cells, we separated equal protein amounts by two-dimensional gel electrophoresis. Gels were stained with Coomassie Blue to demonstrate equal protein amounts in N2a58/22L and N2a58# cells (Figure 1C, left panels). In parallel, gels were blotted onto membranes and incubated with phospho-specific antibodies to detect tyrosine- (Figure 1C, middle panels) or threonine-phosphorylated proteins (Figure 1C, right panels). Interestingly, considerable differences in phosphorylation patterns were observed (Figure 1C, asterisks), while other phosphorylated proteins were not changed in N2a58/22L and N2a58# cells (Figure 1C). These data imply differentially regulated phosphoproteins in response to 22L infection of neuronal cells.

Generally, global detection of phosphorylated proteins is still challenging, as antisera often recognize phosphorylated residues dependent on the surrounding sequence. For a general detection of proteins post-translationally phosphorylated at those sites, we performed a SILAC analysis allowing the identification and relative quantification of differential phosphoprotein regulation. Therefore, N2a58# cells were grown in light isotope containing and N2a58/22L cells in heavy isotope containing medium. Equal amounts of protein lysates were mixed, separated by gel electrophoresis, trypsinized and followed by enrichment of phosphoproteins, which were then analyzed by mass spectrometry. We identified 109 different phosphoproteins of which 105 were also quantified (Tables 1 and 2). We observed 75 proteins with a ratio of identified peptides in N2a58/22L versus N2a58# cells ranging from 0.46 to 0.99 (Table 1). Conversely, 30 phosphoproteins showed a ratio between 1.01 and 1.79 (Table 2). We defined proteins exhibiting a ratio < 0.70 as dephosphorylated proteins and proteins with ratios between 0.70 and 1.40 as proteins, whose phosphorylation was not altered in 22L-infected N2a58/22L cells. Ratios > 1.40 were considered as proteins whose phosphorylation increased upon Scrapie infection.

**Table 1:** Proteins exhibiting decreased phosphorylation in N2a58/22L cells.

**No**.	Uniprot	Protein Names	Ratio^a^	Pept^b^	sequence coverage [%]	PEP^c^	Biological Process
1	P43276	Histone H1.5	**0.46237**	1	13.9	5.61E-16	nucleosome assembly

2	P30681	High mobility group protein B2	**0.48683**	2	11	3.33E-02	genome maintenance; differentiation

3	P11440	Cell division control protein 2 homolog	**0.49428**	5	7.7	3.03E-03	cell cycles; protein phosphorylation

4	P97310	DNA replication licensing factor MCM2	**0.54657**	1	2.4	1.65E-05	cell cycle; nucleosome assembly; transcription

5	P43275	Histone H1.1	**0.56637**	1	19.2	8.42E-04	nucleosome assembly

6	P43274	Histone H1.4	**0.58367**	4	24.2	1.04E-14	nucleosome assembly

7	Q9Z2X1-1	Heterogeneous nuclear ribonucleoprotein F	**0.60054**	2	6.5	5.45E-22	RNA processing

8	P70670	Nascent polypeptide-associated complex subunit alpha, muscle-specific form	**0.60501**	2	1.2	5.07E-11	protein transport; transcription

9	P60843	Eukaryotic initiation factor 4A-I	**0.65781**	5	4.9	5.39E-03	translation

10	P27659	60S ribosomal protein L3	**0.65906**	12	7.7	1.49E-03	translation

11	P28656	Nucleosome assembly protein 1-like 1	**0.70087**	1	7.2	1.16E-05	nucleosome assembly

12	Q62167	ATP-dependent RNA helicase DDX3X	**0.70816**	3	3.3	3.12E-19	putative helicase activity

13	P68040	Guanine nucleotide-binding protein subunit beta-2-like1	**0.71371**	1	7.9	5.15E-08	unknown

14	P47911	60S ribosomal protein L6	**0.71887**	7	12	7.26E-07	translation

15	Q61937	Nucleophosmin	**0.72062**	7	29.8	4.38E-07	cell cycle; nuclear export

16	P15532	Nucleoside diphosphate kinase A	**0.72108**	2	17.8	7.93E-03	NTP biosynthesis; nervous system development

17	O70251	Elongation factor 1-beta	**0.73137**	1	24	2.20E-18	translation

18	Q61656	Probable ATP-dependent RNA helicase DDX5	**0.73832**	4	3.7	1.41E-03	RNA processing; transcription

19	Q9ERK4	Exportin-2	**0.74333**	1	2.1	8.97E-05	cell proliferation; protein transport

20	P09411	Phosphoglycerate kinase 1	**0.74902**	2	6.7	3.40E-03	glycolysis; phosphorylation

21	P48962	ADP/ATP translocase 1	**0.75149**	6	18.8	3.87E-36	transmembrane transport

22	Q9D8N0	Elongation factor 1-gamma	**0.76191**	3	7.8	1.45E-10	translation

23	P49312-2	Heterogeneous nuclear ribonucleoprotein A1	**0.77303**	5	12.1	7.20E-05	alternative splicing; nuclear export/import

24	Q9CZM2	60S ribosomal protein L15	**0.77591**	9	10.3	2.04E-15	translation

25	P97855	Ras GTPase-activating protein-binding protein 1	**0.78021**	2	7.3	1.11E-15	protein transport

26	Q9EQU5-1	Protein SET	**0.79357**	7	7.9	5.77E-12	nucleosome assembly

27	Q7TPV4	Myb-binding protein 1A	**0.79496**	2	1.5	2.39E-11	cytoplasmic transport; transcription

28	P80318	T-complex protein 1 subunit gamma	**0.79504**	1	6.6	2.89E-05	protein folding

29	P25444	40S ribosomal protein S2	**0.79516**	20	12.3	2.99E-04	translation

30	P10126	Elongation factor 1-alpha 1	**0.80203**	5	18.6	1.06E-33	translational elongation

31	P61979-2	Heterogeneous nuclear ribonucleoprotein K	**0.80502**	6	11.9	1.17E-15	RNA processing

32	P07901	Heat shock protein HSP 90-alpha	**0.80637**	2	32.7	1.38E-94	CD8 T-cell differentiation; chaperone activity

33	Q61598-1	Rab GDP dissociation inhibitor beta	**0.81242**	3	5.6	1.93E-05	protein transport; regulation of GTPase activity

34	P54775	26S protease regulatory subunit 6B	**0.81795**	2	7.2	1.59E-09	blastocyst development; protein catabolism

35	Q20BD0	Heterogeneous nuclear ribonucleoprotein A/B	**0.82349**	3	19.3	3.26E-11	nucleotide binding

36	P14206	40S ribosomal protein SA;Laminin receptor 1	**0.8261**	7	20.7	2.57E-17	translation

37	P68134	Actin, alpha skeletal muscle	**0.83457**	10	27.3	4.38E-26	cytoskeleton

38	P80314	T-complex protein 1 subunit beta	**0.83579**	2	11.6	1.05E-21	protein folding

39	P50580	Proliferation-associated protein 2G4	**0.84048**	2	8.1	1.53E-03	rRNA processing; transcription; translation

40	P11983-1	T-complex protein 1 subunit alpha B	**0.84687**	4	8.8	5.73E-23	protein folding

41	P35564	Calnexin	**0.85134**	1	6.3	2.08E-06	protein folding

42	Q8BUP7	Putative uncharacterized protein;26S protease regulatory subunit 6A	**0.85207**	3	7.3	9.72E-03	blastocyst development; protein catabolism

43	P63017	Heat shock cognate 71 kDa protein	**0.85947**	9	32.2	2.16E-96	response to stress

44	Q01768	Nucleoside diphosphate kinase B	**0.86107**	4	17.8	1.35E-04	NTP biosynthesis

45	P62082	40S ribosomal protein S7	**0.86306**	3	10.3	8.39E-03	translation

46	P80315	T-complex protein 1 subunit delta	**0.86423**	1	6.9	3.81E-12	protein folding

47	Q71LX8	Heat shock protein 84b	**0.8654**	4	32	6.13E-136	protein folding; stress response

48	P58252	Elongation factor 2	**0.86672**	2	7.9	3.57E-25	translation

49	P08249	Malate dehydrogenase, mitochondrial	**0.86743**	1	9.2	2.49E-35	glycolysis

50	P70168	Importin subunit beta-1	**0.87257**	2	2.7	3.47E-13	nuclear import

51	P51859	Hepatoma-derived growth factor	**0.87395**	2	12.7	2.95E-07	transcription

52	P14152	Malate dehydrogenase, cytoplasmic	**0.87522**	1	7.8	1.09E-03	glycolysis

53	P80313	T-complex protein 1 subunit eta	**0.89251**	1	8.6	1.22E-39	protein folding

54	P62827	GTP-binding nuclear protein Ran	**0.89794**	3	22.7	2.27E-09	cell cycle; nuclear import; signal transduction

55	P20029	78 kDa glucose-regulated protein	**0.89996**	1	7	4.16E-04	cerebellar Purkinje cell development/organization

56	P56480	ATP synthase subunit beta, mitochondrial	**0.90399**	1	18.5	1.85E-42	proton transport; lipid metabolism

57	P17742	Peptidyl-prolyl cis-trans isomerase A	**0.90719**	10	20.4	4.97E-20	neuron differentiation; protein folding

58	P20152	Vimentin	**0.90814**	10	26.2	2.22E-34	cytoskeleton

59	P09103	Protein disulfide-isomerase	**0.91019**	2	4.7	8.84E-02	redox homeostasis

60	P80317	T-complex protein 1 subunit zeta	**0.91579**	1	10.2	1.49E-02	protein folding

61	Q569Z6	Thyroid hormone receptor-associated protein 3	**0.93507**	3	3.7	4.27E-04	transcription

62	P09405	Nucleolin	**0.93545**	1	12.7	8.06E-34	nucleotide binding

63	Q9D6F9	Tubulin beta-4 chain	**0.93722**	4	23.2	9.43E-32	cytoskeleton

64	Q8C2Q7	Heterogeneous nuclear ribonucleoprotein H1	**0.93795**	3	5.7	2.30E-06	nucleotide binding

65	Q8K019-1	Bcl-2-associated transcription factor 1	**0.94132**	4	5.3	2.22E-05	transcription

66	P62908	40S ribosomal protein S3	**0.94247**	2	9.1	1.74E-06	translation

67	P99024	Tubulin beta-5 chain	**0.9446**	3	36.3	1.15E-54	cytoskeleton

68	P15331-2	Peripherin	**0.95087**	3	17.2	3.41E-40	cytoskeleton

69	P63038-1	60 kDa heat shock protein, mitochondrial	**0.96089**	6	13.8	9.17E-37	T cell activation; interferon production

70	P27773	Protein disulfide-isomerase A3	**0.96765**	1	7.9	1.26E-03	redox homeostasis; apoptosis

71	P16858	Glyceraldehyde-3-phosphate dehydrogenase	**0.96943**	58	23.4	5.69E-66	glycolysis

72	Q3TED3	Putative uncharacterized protein; ATP-citrate synthase	**0.97174**	2	4.3	4.63E-03	acetyl-CoA biosynthesis

73	Q03265	ATP synthase subunit alpha, mitochondrial	**0.98925**	2	13.6	1.81E-22	proton transport; lipid metabolism

74	Q9ERD7	Tubulin beta-3 chain	**0.99071**	2	27.3	5.05E-33	cytoskeleton

75	P17751	Triosephosphate isomerase	**0.9967**	1	11.2	1.52E-04	gluconeogenesis; glycolysis

a. Ratio of N2a58/22L vs. N2a58# cells

b. Number of identified peptides

c. posterior error probability (PEP) estimates the probability of wrong assignment of a spectrum to a peptide sequence

**Table 2:** Proteins exhibiting increased phosphorylation in N2a58/22L cells.

**No**.	Uniprot	Protein Names	Ratio^a^	Pept.^b^	Sequence Coverage [%]	PEP^c^	Biological Process
76	P80316	T-complex protein 1 subunit epsilon	**1.017**	1	6.1	8.63E-18	protein folding
77	Q9CX22	Putative uncharacterized protein; Cofilin-1	**1.0173**	5	29.7	3.72E-41	cytoskeleton; protein phosphorylation
78	P32067	Lupus La protein homolog	**1.02**	2	7.5	1.68E-02	RNA processing
79	A6ZI44	Fructose-bisphosphate aldolase	**1.0328**	8	12.2	2.75E-25	glycolysis
80	P35700	Peroxiredoxin-1	**1.0342**	6	6.7	2.44E-10	proliferation; redox homeostasis; stress response
81	P05202	Aspartate aminotransferase, mitochondrial	**1.0344**	1	12.3	1.04E-11	aspartate biosynthesis; oxaloacetate metabolism
82	Q3TFD0	Serine hydroxymethyltransferase	**1.0425**	3	6.5	2.95E-03	carbon metabolism
83	P63101	14-3-3 protein zeta/delta	**1.0478**	19	19.2	7.09E-27	protein binding
84	Q9CZ30-1	Obg-like ATPase 1	**1.0606**	3	7.1	2.25E-16	ATP/GTP binding; hydrolase activity
85	P06745	Glucose-6-phosphate isomerase	**1.0652**	3	7.7	3.34E-15	angiogenesis; gluconeogenesis; glycolysis
86	Q71H74	Collapsin response mediator protein 4A	**1.0688**	5	6.3	6.94E-12	nervous system development
87	Q6P5F9	Exportin-1	**1.0703**	2	3.6	2.74E-20	nuclear export; centrosome duplication
88	Q01853	Transitional endoplasmic reticulum ATPase	**1.0708**	2	3.7	2.97E-05	apoptosis; retrograde protein transport
89	Q3TCI7	L-lactate dehydrogenase	**1.0746**	4	18.3	1.43E-32	glycolysis
90	P08113	Endoplasmin	**1.08**	1	2.6	1.05E-05	protein folding
91	Q8VC46	Ubc protein;Ubiquitin	**1.0848**	17	30.7	2.18E-06	protein binding
92	A0PJ96	Mtap1b protein	**1.0871**	2	3.2	2.72E-13	cytoskeleton
93	Q61171	Peroxiredoxin-2	**1.0934**	2	9.1	4.16E-04	signal transduction; redox homeostasis
94	Q9WVA4	Transgelin-2	**1.106**	2	11.8	9.37E-05	muscle organ development
95	P52480-1	Pyruvate kinase isozymes M1/M2	**1.1081**	3	22.2	6.30E-39	glycolysis
96	O08709	Peroxiredoxin-6	**1.1204**	5	13.8	1.10E-04	redox homeostasis
97	P17182	Alpha-enolase	**1.128**	11	41.5	6.35E-26	glycolysis
98	P54227	Stathmin	**1.1422**	4	18.1	1.94E-02	cell cycle; cytoskeleton
99	Q922F4	Tubulin beta-6 chain	**1.1427**	1	19.2	1.01E-25	cytoskeleton
100	Q60864	Stress-induced-phosphoprotein 1	**1.1499**	1	3.5	5.12E-05	stress response
101	P52480-2	Pyruvate kinase isozymes M1/M2	**1.2958**	1	19.6	3.40E-33	glycolysis
102	Q920E5	Farnesyl pyrophosphate synthetase	**1.3534**	1	8.2	8.44E-16	cholesterol/isoprenoid biosynthesis
103	P38647	Stress-70 protein, mitochondrial	**1.357**	2	4.6	6.57E-08	nuclear export; protein folding
104	P14824	Annexin A6	**1.6252**	4	7.9	2.47E-08	Ca^2+ ^transport; muscle contraction
105	Q61753	D-3-phosphoglycerate dehydrogenase	**1.7927**	3	8.1	6.28E-15	cell cycle; neural development; serine biosynthesis

a. Ratio of N2a58/22L vs. N2a58# cells

b. Number of identified peptides

c. posterior error probability (PEP) estimates the probability of wrong assignment of a spectrum to a peptide sequence

Among quantified phosphoproteins, we then considered specific phosphosites in selected target proteins, such as Cdc2, stathmin, and cofilin as analyzed by mass-spectrometry (Table 3). An increase of cofilin^S3 ^phosphorylation in N2a58/22L cells was suggested by a ratio 1.63, while the amount of the two tyrosine phosphorylation sites (Y15, Y160) in Cdc2 were decreased upon 22L infection. Stathmin phosphopeptides containing serine 38 were increased, whereas the amount of stathmin phosphopeptides harboring serine 25 in N2a58/22L cells was significantly lower (Table 3).

**Table 3:** Identified phosphorylation sites

Protein names	Ratio (total)^a^	Phosphosite	Ratio (specific phospho-site)^b^
**Cdc2**	0.49428	**Y15**	0.43086
		**Y160**	0.64359

**stathmin**	1.1422	**S25**	1.2155
		**S38**	0.45601

**cofilin**	1.0173	**S3**	1.6328

a. Ratio of phosphorylation N2a58/22L vs. N2a58# cells

b. Ratio of phospho sites in N2a58/22L and N2a58# cells

**Figure 2:**
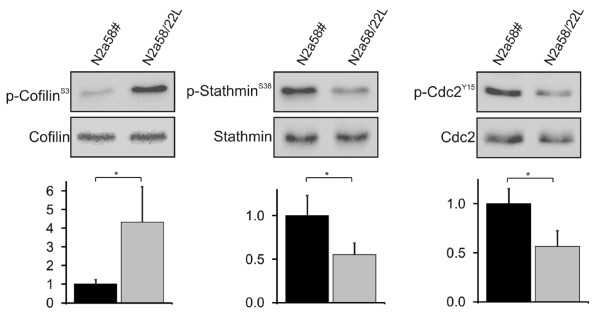
**Specific regulation of cofilin, Cdc2, and stathmin phosphorylation in scrapie-infected neuronal cells**. Cell lysates of N2a58# and 22L-infected N2a58/22L cells were analyzed by Western blot using phospho-specific antibodies to detect p-cofilin^S3^, p-cdc2^Y15^, and p-stathmin^S38 ^(left panels). As loading controls, equal amounts of cofilin, Cdc2 and stathmin were shown. Quantification of intensities of phosphorylation signal was performed by normalizing the corresponding loading control (* *p *< 0.05) (right panels).

To validate the results obtained in the SILAC phosphoproteomic analysis we performed Western blots for cofilin 1, Cdc2, and stathmin using antibodies for the detection of specific phosphosites. As predicted by the SILAC analysis, cofilin 1 phosphorylation was significantly induced in Scrapie-infected N2a58/22L cells compared to PPS-treated N2a58# cells (Figure 2, left panels). Cofilin represents a potent regulator of the actin filaments, which is controlled by phosphorylation of serine 3 mediated through the LIM-kinase 1 (LIMK-1) *in vitro *and *in vivo *[16]. These data support previous studies indicating a direct interaction of PrP^Sc ^with cofilin [17]. Together with our finding that phosphorylation of cofilin is induced in PrP^Sc^-infected neuronal cells; the results indicate a significant role for the protein in neurodegeneration processes. Stathmin acts as an important regulatory protein of microtubule dynamics, which can be directly targeted by Cdc2 [18]. In our analysis, we showed that stathmin^S38 ^phosphorylation was decreased (Figure 2, middle panels), which correlates with the inactivation of Cdc2 in N2a58/22L cells (Figure 2, right panels) implying that there is a functional interaction. Cdc2 is a crucial kinase in starting M phase events during the cell cycle progression and regulates important mitotic structure changes, including nuclear envelope breakdown and spindle assembly [19]. Dephosphorylation of stathmin^S38 ^led to an inhibition of cells at G2/M phase, lack of spindle assembly, and growth inhibition [20,21]. Together with the finding that the prion gene is transcriptionally activated in the G1 phase in confluent and terminally differentiated cells [22], we assume that control of the cell cycle might be important in prion diseases.

Aberrant signal transduction pathways are implicated in many diseases. However, perturbations in phosphorylation-based signaling networks are typically studied in a hypothesis-driven approach. In this study, we performed the first global analysis of the phosphoproteome of scrapie-infected neuronal cells, since the knowledge of PrP-dependent deregulation of the signalling network is poor. SILAC provides a powerful and accurate technique for relative proteome-wide quantification by mass-spectrometry. Its versatility has been demonstrated by a wide range of applications, especially for intracellular signal transduction pathways [23-25]. Since we applied SILAC for the quantitative detection of the phosphoproteome in scrapie-infected neuroblastoma cells, we found 105 different phosphoproteins. Among identified proteins, we validated the regulated phosphorylation of cofilin, stathmin and Cdc2 indicating that the identification of phosphoproteins in scrapie-infected neuronal cells by SILAC is reliable. Future work is necessary to determine whether the identified novel phosphoproteins are involved in prion diseases and if they probably represent sensitive and specific biomarkers for diagnosis or therapeutic intervention strategies.

## Methods

### Cell culture

N2a58/22L cells have been described previously [11] and were kindly provided by Prof. Schätzl (LMU, Munich). Cells were cultured in DMEM containing 10% FCS and 4 mM L-glutamine at 37°C. Cells were treated with 5 μg/ml pentosan polysulfate (Cartrophen Vet, A. Albrecht GmbH + Co. KG, Germany) for two passages, resulting in a stable rescued cell line for more than 15 passages (N2a58# cells). Cell lysates were prepared by scraping cells in lysis buffer containing 150 mM NaCl, 0.5% Triton X-100, 0.5% DOC, 50 mM Tris pH 7.5, 1 mM Na-vanadate, 1 mM Na-molybdate, 20 mM NaF, 10 mM NaPP, 20 mM β-glycerophosphat, 1× protease inhibitor cocktail (Roche, Mannheim, Germany). For digestion with proteinase K (PK) 80 μg protein were treated with 20 μg/ml PK for 30 min at 37°C. PK digestion was stopped by addition of laemmli sample buffer and protein denaturation at 95°C for 7 min.

### Colony assay

The colony assay was performed as previously described with minor modifications [26]. In brief, cells were grown on glass cover slips to confluence using a 24 well plate. The cell layer was soaked in lysis buffer (150 mM NaCl, 0.5% Triton X-100, 0.5% DOC, 50 mM Tris pH 7.5) on a nitrocellulose membrane. After drying for 30 min at room temperature, the membrane was incubated in lysis buffer containing 5 μg/ml proteinase K (PK) for 90 min at 37°C, rinsed twice with water, and incubated in 2 mM PMSF for 10 min. The membrane was shaken in 3 M guanidinium thiocyanate, 10 mM Tris-HCl (pH 8.0) for 10 min, followed by rinsing five times with water. 5% nonfat dry milk in TBS-T was used for blocking for 1 h at room temperature. PrP was detected using an anti-PrP antibody 6H4 (Prionics) and a HRP-conjugated sheep anti-mouse antibody (GE Healthcare).

### SDS-PAGE and Western Blot

Proteins were separated by 12% SDS-PAGE and transferred to polyvinylidene difluoride membranes (PVDF, Millipore) by semidry blotting. PrP was detected using the PrP-specific mouse mAb 8H4 (Alicon AG). For validation of phosphorylated proteins anti-phospho-stathmin (Ser38) (#3426, Cell Signaling Technology), anti-phospho-cdc2 (Tyr15) (#4539, Cell Signaling Technology), and anti-phospho-cofilin (Ser3) antibodies (#3313, Cell Signaling Technology) were used. Antibodies recognizing stathmin (#3352), cdc2 (#9112) and cofilin (#3312) were also obtained from Cell Signaling Technology.

### Two dimensional gel electrophoresis

For 2D electrophoresis 150 μg protein of cell lysates were purified by trichloroacetic acid precipitation and re-suspended in DeStreak Rehydration Solution (Amersham Biosciences) containing 0.5% Bio-Lyte pH3-10 (Bio-Rad Laboratories GmbH, München). The isoelectric focusing was run on IPG strips with a non-linear pH range of 3-10 and a length of 7 cm (Bio-Rad) using the ZOOM^® ^IPGRunner™system from Invitrogen. After focussing strips were equilibrated in 50 mM Tris, 1 mM Urea, 30% Glycerin, 2% SDS, 1% DTT for 25 min and in 50 mM Tris, 1 mM Urea, 30% Glycerin, 2% SDS, 5% Iodacetamid for 25 min. Strips were then separated in 10% SDS-PAGE gels in the second dimension and analyzed by Coomassie staining or immunoblotting using an anti-phospho-tyrosine (sc-7020, Santa Cruz) or an anti-phospho-threonine antibody (#9381, Cell Signaling Technology).

### SILAC phosphoproteomics analysis

SILAC ready-to-use cell culture media and dialyzed FBS were obtained from Dundee Cell Products Ltd, UK. While N2a58# cells were cultured in control SILAC DMEM media containing unlabelled arginine and lysine amino acids (R0K0), N2a58/22L cells were cultured in ready-to-use SILAC DMEM medium containing ^13^C labeled arginine and lysine amino acids (R6K6) for seven cell division cycles. After preparation of cell lysates and measurement of protein concentration, lysates of N2a58# and N2a58/22L cells were mixed in a ratio 1:1. Each sample was reduced in SDS PAGE loading buffer containing 10 mM DTT and alkylated in 50 mM iodoacetamide prior to separation by one-dimensional SDS-PAGE (4-12% Bis-Tris Novex mini-gel, Invitrogen) and visualization by colloidal Coomassie staining (Novex, Invitrogen). The entire protein gel lane was excised and cut into 10 gel slices each. Every gel slice was subjected to in-gel digestion with trypsin [27]. The resulting tryptic peptides were extracted by 1% formic acid, acetonitrile, lyophilized in a speedvac (Helena Biosciences).

### Phosphopeptide enrichment

The lyophilized peptides above were resuspended in 5% acetic acid (binding buffer) and phosphopeptide enrichment was carried out using immobilized metal ion affinity chromatography (IMAC). Immobilized gallium in the Pierce Ga-IDA Phosphopeptide Enrichment Kit was used to enrich for phosphopeptides prior to MS/MS analysis according to the manufacturer's instructions (Thermo Scientific).

### LC-MS/MS

Trypsin digested peptides were separated using an Ultimate U3000 (Dionex Corporation) nanoflow LC-system consisting of a solvent degasser, micro and nanoflow pumps, flow control module, UV detector and a thermostated autosampler. 10 μl of sample (a total of 2 μg) was loaded with a constant flow of 20 μl/min onto a PepMap C18 trap column (0.3 mm id × 5 mm, Dionex Corporation). After trap enrichment peptides were eluted off onto a PepMap C18 nano column (75 μm × 15 cm, Dionex Corporation) with a linear gradient of 5-35% solvent B (90% acetonitrile with 0.1% formic acid) over 65 minutes with a constant flow of 300 nl/min. The HPLC system was coupled to a LTQ Orbitrap XL (Thermo Fisher Scientific Inc) via a nano ES ion source (Proxeon Biosystems). The spray voltage was set to 1.2 kV and the temperature of the heated capillary was set to 200°C. Full scan MS survey spectra (m/z 335-1800) in profile mode were acquired in the Orbitrap with a resolution of 60,000 after accumulation of 500,000 ions. The five most intense peptide ions from the preview scan in the Orbitrap were fragmented by collision induced dissociation (normalised collision energy 35%, activation Q 0.250 and activation time 30 ms) in the LTQ after the accumulation of 10,000 ions. Maximal filling times were 1,000 ms for the full scans and 150 ms for the MS/MS scans. Precursor ion charge state screening was enabled and all unassigned charge states as well as singly charged species were rejected. The dynamic exclusion list was restricted to a maximum of 500 entries with a maximum retention period of 90 seconds and a relative mass window of 10 ppm. The lock mass option was enabled for survey scans to improve mass accuracy [28]. Data were acquired using the Xcalibur software.

### Quantification and Bioinformatic Analysis

Quantification was performed with MaxQuant version 1.0.7.4 [29], and was based on two-dimensional centroid of the isotope clusters within each SILAC pair. To minimize the effect of outliers, protein ratios were calculated as the median of all SILAC pair ratios that belonged to peptides contained in the protein. The percentage variability of the quantitation was defined as the standard deviation of the natural logarithm of all ratios used for obtaining the protein ratio multiplied by a constant factor 100.

The generation of peak list, SILAC- and extracted ion current-based quantitation, calculated posterior error probability, and false discovery rate based on search engine results, peptide to protein group assembly, and data filtration and presentation was carried out using MaxQuant. The derived peak list was searched with the Mascot search engine (version 2.1.04; Matrix Science, London, UK) against a concatenated database combining 80,412 proteins from International Protein Index (IPI) human protein database version 3.6 (forward database), and the reversed sequences of all proteins (reverse database). Alternatively, database searches were done using Mascot (Matrix Science) as the database search engine and the results saved as a peptide summary before quantification using MSQuant http://msquant.sourceforge.net/. Parameters allowed included up to three missed cleavages and three labeled amino acids (arginine and lysine). Initial mass deviation of precursor ion and fragment ions were up to 7 ppm and 0.5 Da, respectively. The minimum required peptide length was set to 6 amino acids. To pass statistical evaluation, posterior error probability (PEP) for peptide identification (MS/MS spectra) should be below or equal to 0.1. The required false positive rate (FPR) was set to 5% at the peptide level. False positive rates or PEP for peptides were calculated by recording the Mascot score and peptide sequence length-dependent histograms of forward and reverse hits separately and then using Bayes' theorem in deriving the probability of a false identification for a given top scoring peptide. At the protein level, the false discovery rate (FDR) was calculated as the product of the PEP of a protein's peptides where only peptides with distinct sequences were taken into account. If a group of identified peptide sequences belong to multiple proteins and these proteins cannot be distinguished, with no unique peptide reported, these proteins are reported as a protein group in MaxQuant. Proteins were quantified if at least one MaxQuant-quantifiable SILAC pair was present. Identification was set to a false discovery rate of 1% with a minimum of two quantifiable peptides. The set value for FPR/PEP at the peptide level ensures that the worst identified peptide has a probability of 0.05 of being false; and proteins are sorted by the product of the false positive rates of their peptides where only peptides with distinct sequences are recognized. During the search, proteins are successively included starting with the best-identified ones until a false discovery rate of 1% is reached; an estimation based on the fraction of reverse protein hits.

Enzyme specificity was set to trypsin allowing for cleavage of N-terminal to proline and between aspartic acid and proline. Carbamidomethylation of cysteine was searched as a fixed modification, whereas *N*-acetyl protein, oxidation of methionine and phosphorylation of serine, threonine and tyrosine were searched as variable modifications.

## Competing interests

The authors declare that they have no competing interests.

## Authors' contributions

WW carried out the experimental work, drafted and wrote the manuscript. PA performed and interpreted the SILAC analysis. JL participated in the design of the study. SW conceived of the study, and participated in its design and coordination and wrote the manuscript. All authors read and approved the final manuscript.
